# Comfort care: the life has always a dignity even if it is very short and its beginning is confused with the end

**DOI:** 10.1186/1824-7288-41-S1-A40

**Published:** 2015-09-24

**Authors:** Franca Sarracino, Immacolata Como, Assia Piccolo, Annalisa Agangi, Antonio Maria Salzano, Francesco Messina, Paolo Puggina

**Affiliations:** 1 Department of Neonatology and Neonatal Intensive Care Unit, “Villa Betania” Evangelical Hospital, Naples 80147, Italy; 2 Department of Obstetrics and Gynaecology, “Villa Betania” Evangelical Hospital, Naples 80147, Italy; 3 Service of Clinical Psychology, “Villa Betania” Evangelical Hospital, Naples 80147, Italy

## 

The comfort-care is an innovative practice introduced recently by the neonatologist Dr. Elvira Parravicini, from Columbia University Medical Center [1]. This practice in a Neonatal Intensive Care Unit (NICU) is a compassionate response that provides families with clear and relevant information and that focuses on the needs of the parents as well as the baby. On the baby side, the need to be kept warm, to be free from hunger, thirst or pain are the cornerstones of the program. On the family side, it is a viable option of care for fetus/neonates who are suffering from life-limiting conditions which takes in to account the emotional needs of the family at this difficult time [2]. When we are not able to ensure medical treatments aimed at curing the disease, we can help in a different way. Palliative care planning involves multidisciplinary team planning with professionals from (1) gynecologist (2) midwife, (3) neonatologist, (4)pediatric nurse, (5) psychologist. The multidisciplinary approach is pointed on the satisfaction of “essential needs” of both the family and the baby starting from the pre birth care to the post death care. The team's goals cover practical aspects of infant care, including pain relief, symptom relief, comfort and dignity, the management of prognostic uncertainties, but also the provision of support to families during the pregnancy, their baby's illness and afterwards when coming to terms with his loss. The target population is all infants with a life-limiting conditions (trisomy 13, trisomy 18, bilateral renal agenesis or anencephaly, etc.) for whom a decision has been made to not interrupt the pregnancy [1]. The care planning is very flexible and continuously consideres parents’ personal and/or spiritual wishes, moreover it is continuously reviewed in the best interests of the baby. Multidisciplinary discussions and decision making involving the parents and the team to plan the management are essential. At least also the staff is provided with informal and formal support during the period of providing palliative care [3,4].

## References

[B1] Parravicini E Is “comfort” care a “medical” care? Observations on a neonatal population J Med Pers 2012 10 1 41 45 10.1007/s12682-011-0108-4

[B2] Whool C Systematic review of the literature. Parental outcomes after diagnosis of fetal anomaly Adv Neonatal Care 2011 11 182 192 10.1097/ANC.0b013e31821bd92d 21730912

[B3] Whitfield JM Siegel RE Glicken AD Harmon RJ Powers LK Goldson EJ The application of Hospice Concepts to Neonatal Care Am J Dis Child 1982 136 421 424 6177238 10.1001/archpedi.1982.03970410039009

[B4] Paedriatr Child Health 2001 6 469 477

